# Population dynamics in the Japanese Archipelago since the Pleistocene revealed by the complete mitochondrial genome sequences

**DOI:** 10.1038/s41598-021-91357-2

**Published:** 2021-06-13

**Authors:** Fuzuki Mizuno, Jun Gojobori, Masahiko Kumagai, Hisao Baba, Yasuhiro Taniguchi, Osamu Kondo, Masami Matsushita, Takayuki Matsushita, Fumihiko Matsuda, Koichiro Higasa, Michiko Hayashi, Li Wang, Kunihiko Kurosaki, Shintaroh Ueda

**Affiliations:** 1 Department of Legal Medicine, Toho University School of Medicine, Tokyo, Japan; 2 Department of Evolutionary Studies of Biosystems, SOKENDAI (The Graduate University for Advanced Studies), Hayama, Japan; 3 Advanced Analysis Center, National Agriculture and Food Research Organization, Tsukuba, Japan; 4 Department of Anthropology, National Museum of Nature and Science, Tokyo, Japan; 5 Department of Archaeology, Faculty of Letters, Kokugakuin University, Tokyo, Japan; 6 Department of Biological Sciences, Graduate School of Science, The University of Tokyo, Tokyo, Japan; 7 The Organization of Anthropological Research, Tokyo, Japan; 8 Graduate School of Medicine, Kyoto University, Kyoto, Japan; 9 Department of Genome Analysis, Institute of Biomedical Science, Kansai Medical University, Hirakata, Japan; 10 School of Medicine, Hangzhou Normal University, Hangzhou, China

**Keywords:** Anthropology, Biological anthropology

## Abstract

The Japanese Archipelago is widely covered with acidic soil made of volcanic ash, an environment which is detrimental to the preservation of ancient biomolecules. More than 10,000 Palaeolithic and Neolithic sites have been discovered nationwide, but few skeletal remains exist and preservation of DNA is poor. Despite these challenging circumstances, we succeeded in obtaining a complete mitogenome (mitochondrial genome) sequence from Palaeolithic human remains. We also obtained those of Neolithic (the hunting-gathering Jomon and the farming Yayoi cultures) remains, and over 2,000 present-day Japanese. The Palaeolithic mitogenome sequence was not found to be a direct ancestor of any of Jomon, Yayoi, and present-day Japanese people. However, it was an ancestral type of haplogroup M, a basal group of the haplogroup M. Therefore, our results indicate continuity in the maternal gene pool from the Palaeolithic to present-day Japanese. We also found that a vast increase of population size happened and has continued since the Yayoi period, characterized with paddy rice farming. It means that the cultural transition, i.e. rice agriculture, had significant impact on the demographic history of Japanese population.

## Introduction

The Japanese Archipelago located in the boundary of the Eurasian, the North American, the Pacific and the Philippine Sea Plates was created by separating from the Asian continent by 15 million years ago. Archaeological evidence indicates the first appearance of people in the Japanese Archipelago during the late Pleistocene between 40,000 and 30,000 years ago. The prehistoric era after the Palaeolithic era is typically split into two contrasting periods, Jomon and Yayoi. The Jomon period is characterized by hunter-gatherer subsistence that persisted for more 10,000 years. The name of Jomon derived from the distinctive rope-pattern decorations on the pottery. The presence of pottery in Jomon period shows that making pottery was not just unique to agricultural peoples. On the other hand, the Yayoi period is characterized as paddy rice farming. And it is thought that this style of rice production was introduced into the Japanese Archipelago by migrants from mainland Asia and that the agricultural migrants came to the Japanese Archipelago in and after the Yayoi period. Since the middle nineteenth century several hypotheses have been proposed in regard to the population history of present-day Japanese, but it is now popularly accepted that the present-day Japanese consists of at least two ancestries, one from southeast Asia-originated Jomon people and the other from northeast Asia-originated Yayoi people, although scarce knowledge of Palaeolithic people^1–4^.

The Japanese Archipelago is widely covered with acidic soil made of volcanic ash, making ancient DNA studies challenging. This is the first report of Palaeolithic mitogenome (mitochondrial genome) sequence in the Japanese Archipelago. Here we investigate the population dynamics in the Japanese Archipelago using complete mitogenome sequences from the Palaeolithic, Jomon, Yayoi and present-day Japanese, and find continuity in maternal gene pool among them from the point of view of haplogroup diversity. Through demographic analysis using over 2,000 present-day Japanese, we observe drastic population explosion across cultural transition from hunter gathering to farming. This was a period in which the environment is known to have undergone short, rapid fluctuations in temperature. During the transition from the Last Glacial Period (LGP) to the present Holocene, there was also a temporary return to glacial conditions, known as the Younger Dryas. This rapid climate change has been shown to be strongly associated with the widespread extinction of megafauna^5,6^.

Mitochondrial Haplogroup M is frequently observed in present-day Asian populations but is not found in present-day European populations^7,8^, while analysis of mitochondrial DNA from archaeological human remains dating to the Late Pleistocene has identified individuals belonging to haplogroup M that populated Europe before the LGP. This indicates drastic change in the maternal gene pool^9–12^. By obtaining complete mitogenome sequences, we here investigate genetic relationships among the Palaeolithic, the following hunter-gathering Jomon and agricultural Yayoi people, and furthermore disclose the past demographic history of present-day Japanese population. In case when we found a mitochondrial DNA whose haplogroup is not found in the present-day Asian population, there would be a possibility of drastic change of maternal gene pool as observed in Europe. But in case when we found a mitochondrial DNA whose sequence is closely related to the mitochondrial DNA found in present-day Asian population, there would be less possibility of drastic change of maternal gene pool, namely population continuity.

## Results and discussion

### Mitogenome of the Palaeolithic remains

**Figure 1:**
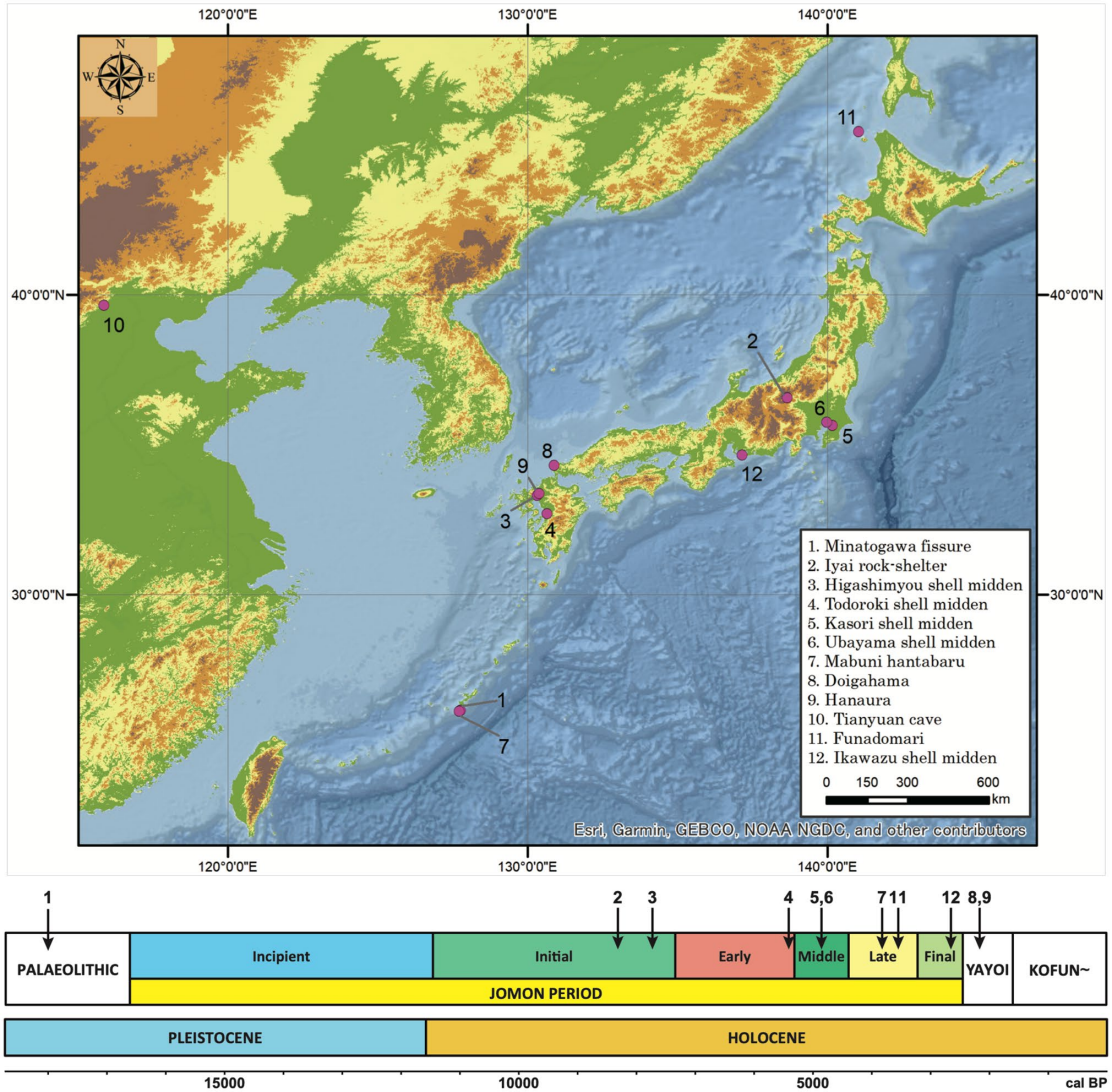
Late Pleistocene and Holocene archaeological sites in the Japanese Archipelago and mainland China. Only the sites where mitogenome sequences from human remains used in this study are shown. The known dates for each site are shown below. This figure was generated by Adobe Illustrator 24.1 (Windows) and ArcGIS Desktop 10.7.1 (https://www.esrij.com/). The data was obtained from Consultative Group on International Agricultural Research Consortium for Spatial Information (CGIAR-CSI, https://cgiarcsi.community/), CGIAR-CSI SRTM 90 m Database ^53^ (http://srtm.csi.cgiar.org) and Ocean/World_Ocean_Base of ArcGIS map service (http://services.arcgisonline.com/arcgis/services).

**Figure 2:**
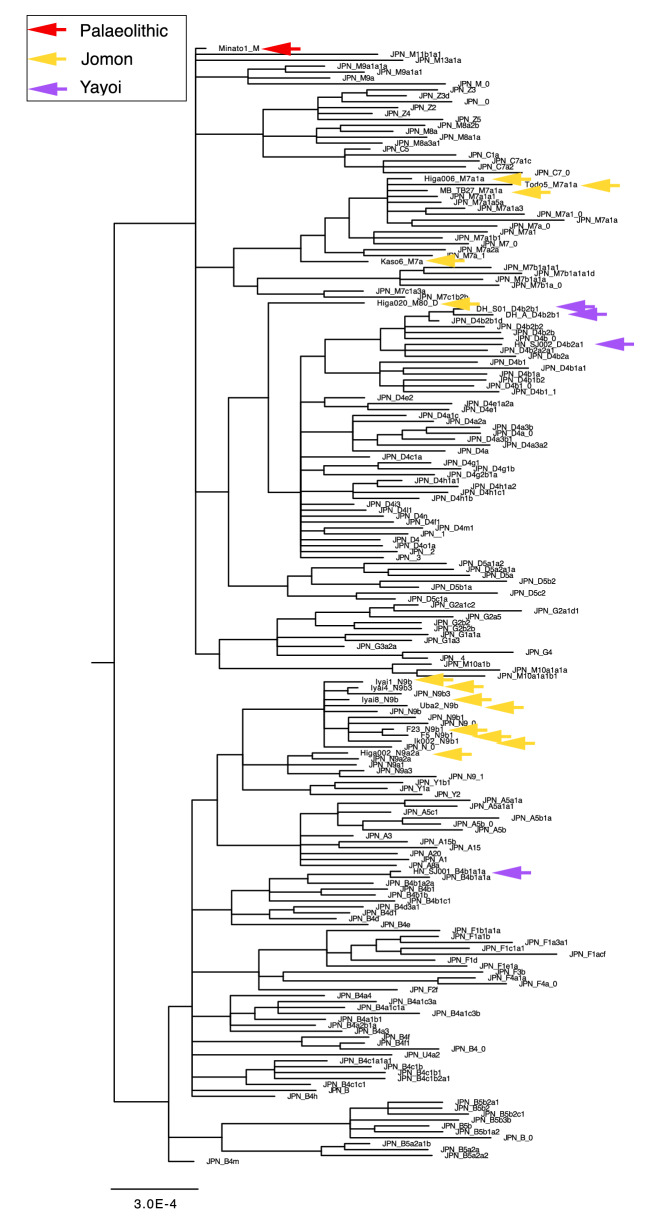
Bayesian phylogenetic tree of complete mitogenome sequences of 18 ancient and 171 present-day individuals in the Japanese Archipelago. Coloured arrows show the positions of ancient individuals; red, yellow, and purple for Palaeolithic, Jomon, and Yayoi, respectively. The tree was rooted with mitogenome haplogroup L0a (accession number: EF184601.1). The estimated haplogroups for newly determined ancient samples are Minato1:M, Iyai1:N9b, Iyai4:N9b3, Iyai8:N9b, Higa002:N9a2a, Higa006:M7a1a, Higa020:M80'D, Todo5:M7a1a, Kaso6:M7a, Uba2:N9b, MB-TB27:M7a1a, DH-S01:D4b2b1, DH-A:D4b2b1, HN-SJ001:B4b1a1a and HN-SJ002:D4b2a1, respectively (Table 1 and Supplementary Table S3).

**Figure 3:**
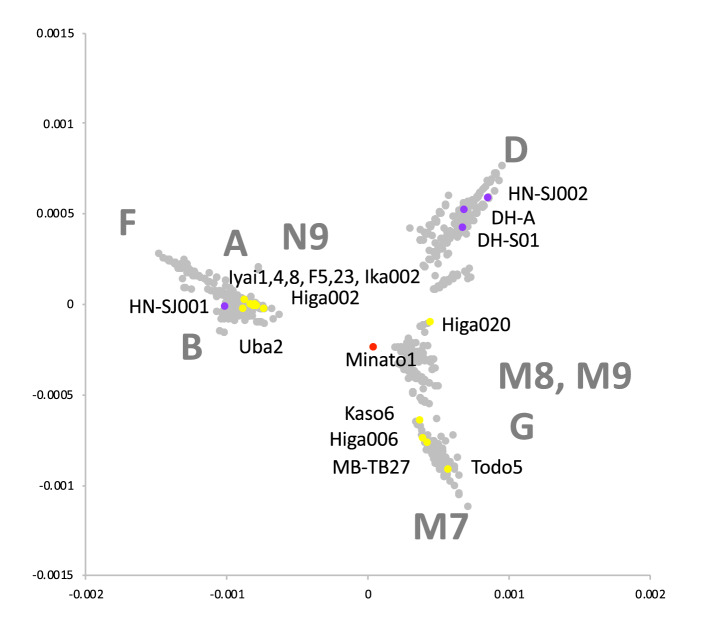
Multi Dimensional Scaling (MDS) plot of whole coding sequences of mitogenome of 1 Palaeolithic (red), 13 Jomon (yellow), 4 Yayoi (purple), and 2,062 present-day (grey) individuals in the Japanese Archipelago. For present-day Japanese, assigned haplogroups were shown.

**Figure 4:**
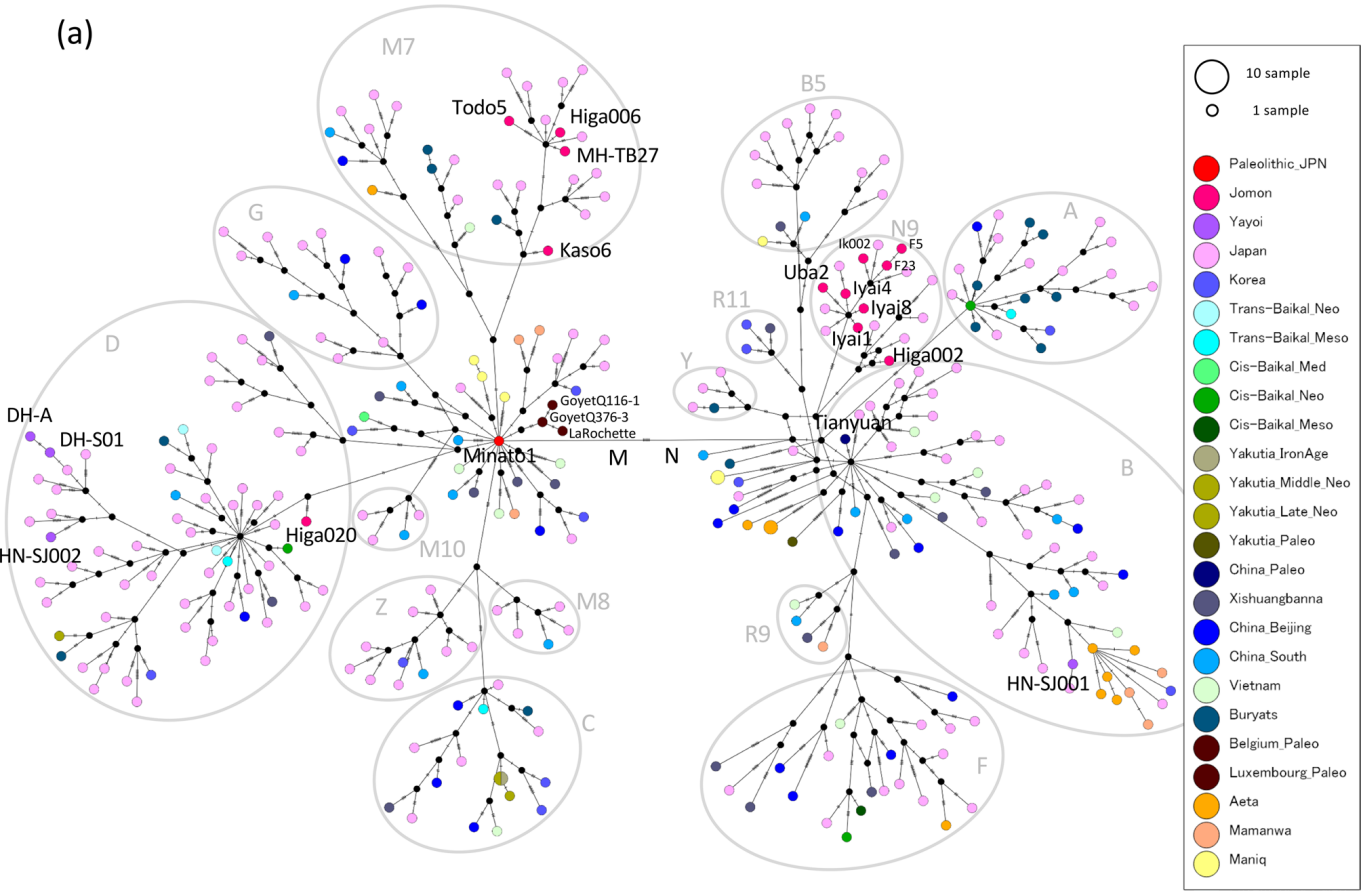

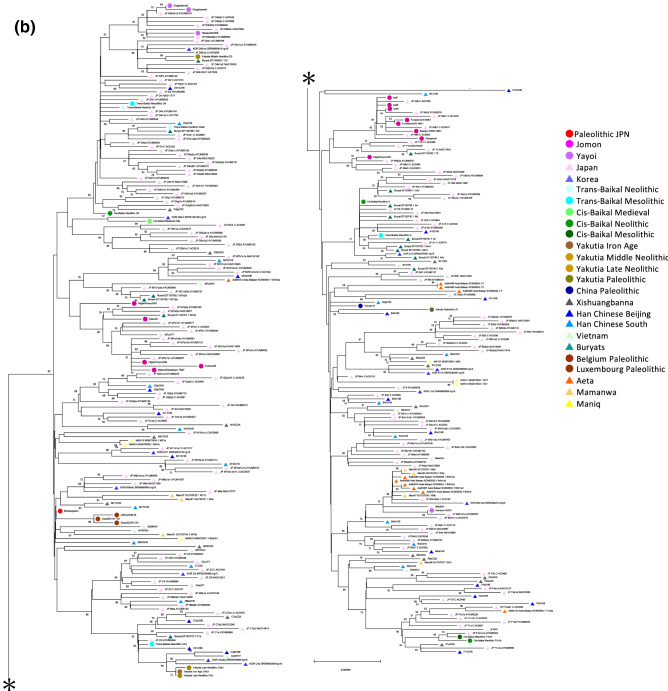
(**a**) A median-joining network for whole coding mitogenome of 37 ancient individuals and 287 present-day Asians belonging to macrohaplogroups M and N. Number of mutations was shown as hatch marks on branches. (**b**) The phylogenetic tree inferred using the Neighbor-Joining method for world-wide 37 ancient individuals and 287 present-day Asians belonging to macrohaplogroups M and N. The samples used here belong to either of macrohaplogroup M (including C, D, G, M7, M8, M10, and Z), and macrohaplogroup N (including A, B, B5, F, N9, R9, R11, and Y). The evolutionary distances for whole coding region of mitochondrial genome, totally 14,918 bp, were computed using the Maximum Composite Likelihood method. The percentage (50% and greater) of replicate trees in which the associated taxa clustered together in the bootstrap test (1,000 replicates) are shown next to the branches. All positions containing gaps and missing data were eliminated (complete deletion option).

We successfully determined a highly accurate complete mitogenome sequence of 20,000-year-old Minatogawa 1 (Minato1), a plausible direct descendant of the initial migration into the Japanese Archipelago (Fig. 1, Table 1 and Supplementary Table S1). Its sequence obtained with average depth of 52 was classified into haplogroup M, and carries no substitutions that are defining subgroups of haplogroup M. Haplogroup M is found at high frequency in present-day Asians, Australasians, and indigenous Americans^13–16^. The sequence of this ancestral type of haplogroup M is not seen in any of 2,062 present-day Japanese samples newly determined in this study, 672 present-day Japanese^15^, 21,668 Han Chinese^17^. Figure 2 shows a Bayesian phylogenetic tree of mitogenome of 18 ancient and 171 present-day individuals in the Japanese Archipelago. Figure 3 shows a Multi Dimensional Scaling (MDS) plot of mitogenome of 1 Palaeolithic, 13 Jomon, 4 Yayoi, and 2,062 present-day individuals in the Japanese Archipelago. Supplementary Figure S1 shows a MDS plot including present-day East Asian samples. These results show that Minato 1 does not make clear cluster with any of the other samples, suggesting the Minato 1 is not directly related to the Jomon, Yayoi, and present-day Japanese. But it located near the root of haplogroup M. This suggests that Minato1 belongs to the ancestral population of present-day Japanese but also to the ancestral population of present-day East Asians. A similar case was reported in Asian mainland, a 40,000-year-old Tianyuan individual excavated from northern China. It was reported to be the mitochondrial sequence of ancestral-type haplotype B having four singletons (private mutations), i.e. a common ancestor of present-day mitogenome belonging to haplogroup B^18,19^. Due to fluctuating global warming, Palaeolithic period from the LGP is supposed to be a difficult time to survive in^5,6^, and changes in the gene pool are expected to occur various populations all across the world. However, the results of phylogenetic network and Neighbor-Joining phylogenetic tree including Minato1 and Tianyuan individuals (Fig. 4a,b) indicate that drastic changes in maternal gene pool during the LGP had not occurred in East Asia because it shows that both Minato 1 and Tianyuan belong to the ancestral population of present-day East Asians.

**Table 1:** Late Pleistocene and Holocene human remains of the Japanese Archipelago used in this study.

Individual ID	Era	Site	Approximate date	mtDNA haplogroup	References
Minato1	Paleolithic	Minatogawa fissure	19,900 cal BP	M	This study
Iyai1	Initial Jomon	Iyai rock-shelter	8,300–8,200 cal BP	N9b	Our study (Mizuno et al. 2020) ^31^
Iyai4	Initial Jomon	Iyai rock-shelter	8,300–8,200 cal BP	N9b3	Our study (Mizuno et al. 2020) ^31^
Iyai8	Initial Jomon	Iyai rock-shelter	8,300–8,200 cal BP	N9b	Our study (Mizuno et al. 2020) ^31^
Higa002	Initial Jomon	Higashimyou shell midden	–	N9a2a	This study
Higa006	Initial Jomon	Higashimyou shell midden	7,934–7,792 cal BP	M7a1a	This study
Higa020	Initial Jomon	Higashimyou shell midden	–	M80'D	This study
Todo5	Early Jomon	Todoroki shell midden	6,210–6,094 cal BP	M7a1a	This study
Kaso6	Middle Jomon	Kasori shell midden	–	M7a	This study
Uba2	Middle Jomon	Ubayama shell midden	–	N9b	This study
MB-TB27	Late Jomon	Mabuni hantabaru	–	M7a1a	This study
F5	Late Jomon	Funadomari	3,800–3,500 cal BP	N9b1	Kanzawa-Kiriyama et al.^51^
F23	Late Jomon	Funadomari	3,800–3,500 cal BP	N9b1	Kanzawa-Kiriyama et al.^51^
Ik002	Final Jomon	Ikawazu shell midden	2,720 cal BP	N9b1	McColl et al.^52^
DH-S01	Middle Yayoi	Doigahama	2,306–2,238 cal BP	D4b2b1	This study
DH-A	Middle Yayoi	Doigahama	–	D4b2b1	This study
HN-SJ001	Middle Yayoi	Hanaura	–	B4b1a1a	This study
HN-SJ002	Middle Yayoi	Hanaura	–	D4b2a1	This study

### Mitogenome of the hunting-gathering Jomon remains

We further successfully determined highly accurate complete mitogenome sequences with average depths of coverage from 11 to 1,577 using 8,300- to 3,700-year-old Jomon individuals (10 individuals from six archaeological sites, Fig. 1, Table 1 and Supplementary Table S1). All of the Jomon individuals fall into the same clusters with present-day Japanese in phylogenetic network, phylogenetic trees and MDS plot (Figs. 2, 3, 4). This shows population continuity from Jomon period to present-day in the Japanese Archipelago, meaning no dynamic change in the maternal gene pool of people living in the Japanese Archipelago from the Palaeolithic to Neolithic periods. The results also show that most of the Jomon individuals in our collection are of haplogroups M or N, and many of them are of sub-haplogroups M7a or N9b in the narrow taxonomic group (Table 1 and Supplementary Table S1). This is consistent with the previous observations from PCR-based typing^20,21^. Both haplogroups M7a and N9b characteristic of Jomon individuals have been inherited by the present-day Japanese individuals; 7.9% and 1.3% among the present-day Japanese population, respectively (Supplementary Table S2).

### Mitogenome of the farming Yayoi remains

In addition, we successfully determined highly accurate complete mitogenome sequences of four individuals from Yayoi period with average depths of coverage from 13 to 5,891 (each two individuals from the Hanaura and Doigahama Yayoi sites, respectively. Figure 1, Table 1 and Supplementary Table S1). The Yayoi period started by the lifestyle of paddy rice farming that was brought into Japanese Archipelago by Yayoi migrants. The results show that three of the Yayoi individuals belong to haplogroup D4 (Table 1). D4 is the most common haplogroup in present-day Japanese (34.3%) (Supplementary Table S2), which is also common throughout East Asia^15,17^. Like the Jomon individuals, all of the Yayoi individual fall into any of the clusters of the phylogenetic network, MDS, Bayesian and Neighbor-Joining phylogenetic trees that are constructed together with the present-day Japanese people, although the Jomon and Yayoi people have some mitochondrial sub-haplogroups characteristic to each of them (Figs. 2, 3, 4). Combining with the results of Minato 1 and Jomon samples, it is shown that there is at least some population continuity from the late Pleistocene to present-day human populations in the Japanese Archipelago.

### Estimating past demographic trends

**Figure 5:**
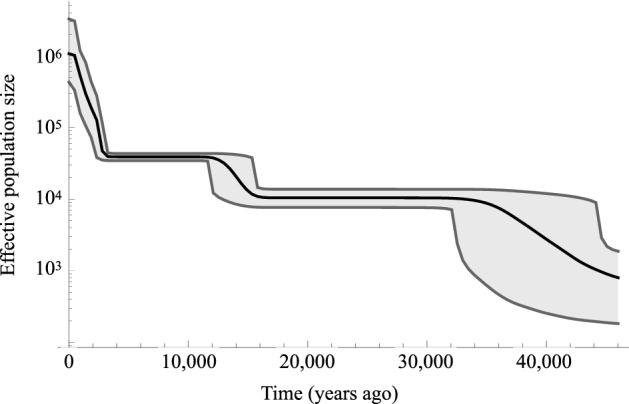
Bayesian Skyline Plot (BSP) of 2,062 present-day Japanese. Effective population size (y axis, log-scale) was plotted against time (x axis, years ago). Solid black line shows the mean effective population size from the posterior distribution. Two grey lines and grey coloured region within them show the 95% posterior density intervals.

Our results show that the gene pool of the present-day Japanese has been established, swallowing all of the genetic diversities of the people living in the Japanese Archipelago during more than 10,000 years, starting from the Palaeolithic period through the hunter-gatherer Jomon and farming Yayoi periods. Bayesian-Skyline Plot (BSP) analysis using 2,062 present-day Japanese found three large population increases, at 45,000–35,000 BP, 15,000–12,000 BP, and 3,000 BP (Fig. 5). These correspond with a rise in temperatures seen in the Late Pleistocene, the dawn of agriculture in East Asia, and the beginning of the Yayoi period, respectively. In a recent study relating to East Asia, demographic history was referred from complete mitogenome sequences of 21,668 present-day Han Chinese individuals^17^. The results showed that there was a population increase towards the end of the LGP around 45,000–35,000 BP, followed by another, more rapid increase during the Neolithic period 15,000–12,000 BP. The first two population increases are common among the present-day Japanese and Chinese populations, whereas the third one can be seen uniquely in the present-day Japanese. And the third phenomenon unique to the Japanese Archipelago, which took place after beginning of the Yayoi period, did great contribution to population size of the present-day Japanese. Combined with them, the first two population increases seen in the present-day Japanese populations should have mainly reflected ones occurred before the ancestors of Yayoi people migrated to the Japanese Archipelago, namely the increases taken place in the Asian mainland. It is easily expected that the Yayoi migrant bringing rice paddy farming should have a significant impact on the number of population and population structure in the Japanese Archipelago. It is suggested that Holocene climate shifts happened 2.8 ka or 4.2 ka BP events affected the population of Korean Peninsula and drove migration to Japanese Archipelago^22,23^. Further population increase afterwards could be related to the introduction of ironware into the Japanese Archipelago, which allowed for more efficient rice paddy farming and a more stable food supply^24^. Given all the findings obtained, our results show that genetic makeup of the present-day Japanese populations is built by migration events of Yayoi farmers and subsequent multiple migrations from the Asian mainland. But we cannot ignore contribution of the Jomon people to the present-day Japanese population structure.

We show (a) there were continuity in the maternal gene pool since the initial wave of migration into the Japanese Archipelago of Palaeolithic period from the point of view of haplogroup diversity, (b) ancestors of the present-day Japanese had experienced three large population increases, but the first two of them mainly occurred in the Asian mainland. The third increase was a sharp one occurred in relatively short period, (c) gene pool of the Jomon hunter-gatherers has survived, even after migration of the Yayoi farmers and its subsequent population explosion.

The following key questions remains open such as whether the migrating Yayoi farmers genetically mixed with the indigenous Jomon hunter-gatherers at each stage after the Yayoi migration (and, if so, what type of mix occurred)^25–27^. Our study includes only mitogenome and the number of samples is limited. Therefore, to reach more solid or concrete conclusion, we need to reveal more genome information of ancient people then we can obtain clearer evidence on the presence of friendly relationships between Jomon hunter-gatherers and Yayoi farmers. Further mitogenome investigation of other Palaeolithic remains and nuclear genome analysis of Minato 1 should give us more clues and details to disclose human history in Japanese Archipelago.

## Materials and methods

Approval for the present study was provided by the Ethics Committee of Toho University School of Medicine (A20110_A18099_A18056, A19095). Approval was also provided by the Ethics Committee of Department of Evolutionary Studies of Biosystems, SOKENDAI; The Graduate University for Advanced Studies (SKD2019HG001). The Ethics Committee of Kyoto University Graduate School and Faculty of Medicine (G0751-4), the Ethical Review Board of the Nagahama Study, and the Nagahama Municipal Review Board of Personal Information Protection approved all study procedures. All methods were performed in accordance with the relevant guidelines and regulations.

### Ancient samples

Bones and teeth from human remain representing 15 individuals from nine archaeological sites were sampled for ancient genome analyses; Minatogawa fissure and Mabuni hantabaru (Okinawa Pref.), Iyai rock-shelter (Gunma Pref.), Higasimyou and Hanaura (Saga Pref.), Todoroki shell midden (Kumamoto Pref.), Kasori shell midden and Ubayama shell midden (Chiba Pref.), Doigahama (Yamaguchi Pref.) (Supplementary Table S1, Fig. 1). Dates of five samples are available, the others are referenced on pottery typological chronology. Minato1 indicates many archaic morphological features, such as a thick and inferiorly wide skull vault, a small frontal bone with extremely developed temporal fossae, and a rugged facial profile. It is reasonable to suppose that it was remnants of the earliest *Homo sapiens* in East Asia, who somehow migrated to Okinawa Islands around 30,000 or 40,000 years ago and survived at least until around 20,000 years ago, keeping these archaic features^28,29^.

### DNA extraction, NGS library preparation, enrichment of the ancient mitochondrial genome and sequencing

DNA was extracted from bones and teeth, and NGS library was constructed according to our previous methods^30^ with slight modifications as follows^31^. After exhaustively brushing to eliminate dirt and exogenous contaminants, the outer bone surface was mechanically removed with a sanding machine (Dremel) to further remove surface contaminants. The clean bone was cut into pieces of approximately 1–2 cm^3^ using an electric drill cutter (Dremel). The bone fragments were cooled with liquid nitrogen, and fine powdered bone was obtained by grinding bone fragments in a mill (Multi-beads Shocker MB601U, YASUI KIKAI). During all steps of DNA extraction, NGS library preparation, and enrichment, we took all possible precautions to guard against contamination. Experiments were performed in a laboratory that is exclusively dedicated to ancient DNA work and is physically isolated from other molecular work laboratories. All manipulations were performed in a laminar flow cabinet routinely irradiated with UV light. Frequent surface cleaning was routinely performed before and after working. A facemask, head cap, and clean laboratory coat were always worn, and gloves were frequently replaced. All the procedures were conducted using new sterilized disposable tubes and filter pipette tips. All non-disposable glass and metallic materials were dry-heat sterilized at 160 °C for 2–4 h. The powdered samples (10 to 200 mg) were digested in 0.5 M EDTA (pH 8.0) for 2 h at 56 °C in a rotating hybridization oven, and the supernatant was removed by centrifugation. This decalcification step was repeated three times in total. DNA was extracted with phenol:chloroform:isoamyl alcohol (25:24:1), followed by extraction with an equal volume of chloroform. After centrifugation, the aqueous solution was removed and subsequently concentrated by centrifugation dialysis using an Amicon Ultra-15 30 kDa centrifugal filter (Merck Millipore) to a final volume of 200 μL. The DNA solution was purified with silica-based MiniElute spin columns (Qiagen) according to manufacturer's protocol. The obtained DNA was quantified using Quant-iT dsDNA HS assay kit (Thermo Fisher Scientific). Using the obtained DNA, we prepared single- or double-strand NGS libraries. For some samples, PreCR Repair Mix (New England BioLabs) treatment was done. Libraries were enriched for human mitochondrial genomes, using in-solution target enrichment (SureSelect, Agilent Technologies). The libraries were pooled sequenced on illumina MiSeq or HiSeq platform 1500 according to the manufacturer's instructions.

### NGS data processing and ancient DNA authentication

Raw sequencing reads were trimmed by removing adapter sequences and low-complexity sequences with fastp ver. 0.20.0 (parameters: -l 30 -y –detect_adapter_for_pe)^32^. The trimmed reads were mapped against the human mitogenome reference sequence rCRS^33^, using the Burrows-Wheeler Alignment tool (BWA)^34^ with optimal parameters for ancient DNA (parameters: -l 1024 -n 0.01 -o 2)^35^. Reads with a mapping quality lower than 30 were filtered out and high-quality mapped reads were retained using samtools ver. 1.9^36^. To minimize the effects of nucleotide mis-incorporations on building a consensus mitogenome sequence, the first two bases on each end of the read were clipped with BamUtil ver. 1.0.14^37^ according to our previous bioinformatics procedure^38^ Deamination is considerably repaired by using PreCR Repair Mix, but even if it is repaired, it is difficult to completely repair it. Because the damage still remains, both ends are clipped. Then, reads with a significant hit (E-value <  = 1e−15) to non-human genomes (e.g., fungal or bacterial genomes) were identified by BLASTN and then were also filtered out. In addition, reads mapped to human nuclear genome sequences of hg19 were also removed as numts (nuclear copies of mitogenome). Finally, consensus sequences were built using MitoSuite^39^, and their mitogenome haplogroup assignments were called with HaploGrep2^40^. We checked variants using IGV software^41^ based on PhyloTree Build 17 (http://www.phylotree.org/)^42^. To assess whether the results could be affected by present-day human contamination, sequences with ancient DNA specific cytosine to thymine substitutions (C to T) at the 5’ or 3’ molecule end were selected and analysed separately (Supplementary Fig. S2). DNA contamination was estimated using MitoSuite ver.1.0.9^39^.

### Present-day Japanese samples

Present-day Japanese examined in this study are participants of the baseline survey in the Nagahama Study. The Nagahama Study is an ongoing community-based cohort study conducted by the Kyoto University Graduate School of Medicine and Nagahama City. The participants are members of the general population living in Nagahama City, a rural city of 125,000 inhabitants in Shiga Prefecture located in central Japan, aged 30 to 74 years old.

### NGS data processing of present-day Japanese mitogenome DNA

Whole genome sequencing of present-day Japanese was conducted using the Illumina HiSeq X Ten sequencer (Illumina Inc., San Diego, CA, USA). After aligning the sequence reads onto the reference genome (GRCh37/hg19) using the Burrows-Wheeler Aligner with default parameters, according to the developer's manuals^34^. Then, duplicate reads were marked using Picard. A total of 2,062 subjects were included in the present study, of which complete mitogenome sequences are available with high depth of coverage. Finally, consensus sequences were built from the BAM files of chrM using MitoSuite^39^, and their mitogenome haplogroup assignments were called with HaploGrep2^40^ (Supplementary Table S2). We checked variants using IGV software^41^ based on PhyloTree Build 17^42^.

### Statistical phylogenetic analyses

To produce Bayesian phylogenetic tree, we used mrbayes v. 3.2^43^. “GTR + I + Γ” model was used for substitution model. Model was selected by Maximum Likelihood fits function in MEGA X^44^. We set the Markov Chain Monte Carlo (MCMC) chain length to 3.0 × 10^6^ with 7.5 × 10^5^ burn-in steps to collect sufficient samples for estimation. In this Bayesian tree, we selected 171 present-day Japanese mitochondrial genome sequences to cover major haplotypes belonging macro haplogroups M and N (see Supplementary Table S3 for the list of sequence used) for the ease of computation. Our newly determined 15 ancient mitochondrial genome sequences were aligned together and included in the tree estimation. The tree was estimated by using haplogroup L0a (accession number: EF184601.1) as an outgroup. For MDS, we used 2,062 complete mitogenome sequences from Japanese population, which were produced in this study. Sequences without “N” nucleotide were used. We used mafft v7.471 to align these 2,062 sequences with rCRS sequence^45^. Mutations were scored relative to the rCRS and we extracted the coding regions. The 15 ancient mitochondrial genome sequences produced in this study were added to this multiple sequence alignment. MDS was performed on R software with cmdscale() function^46^. We used p-distance based on the number of differences on nucleotide sequences for MDS. We used MEGAX for obtaining p-distances^44^. A median-joining Network^47^ was constructed by using PopART v1.7^48^ with a parameter “epsilon = 0”. We selected 287 modern Asian mitochondrial genome sequences to cover major haplotypes belonging macro haplogroups M and N. Also, 22 ancient mitochondrial genomes available were obtained. Together with above dataset, our newly determined 15 ancient mitogenome sequences were aligned, and then, variant sites in coding region of mitogenome was used as an input for the network analysis (see Supplementary Tables S3 and S4 for the lists of sequence used). The resultant network was manually modified to improve visibility on PopART.

### Bayesian skyline plot (BSP)

To obtain BSP, we used BEAST v2.5.0 suite^49^. We applied the general time reversible model mutation model using gamma-distributed rate. A clock model was used assuming a log-normal distribution. We set the Markov Chain Monte Carlo (MCMC) chain length to 1 × 10^10^ with 1 × 10^9^ burn-in steps to collect sufficient samples for parameter estimation. We performed the MCMC simulation twice independently to confirm that the simulation converged to the same state. The time was scaled by the number of mutations for BSP construction. To estimate time in year, we assumed a molecular clock of 2.77 × 10^–8^ and applied the correction method of Soares et al.^8, 50^. To scale the effective population size (Ne), we assumed a generation time of 25 years. The simulation results were analysed using Tracer v1.7.1 (http://tree.bio.ed.ac.uk/software/tracer/).

## Supplementary Information

Supplementary Information.

## Data Availability

Sequencing data produced in this study have been deposited in the GenBank (accession no. LC521960- LC521962, LC597326-597337) and Human Genetic Variation Database (accession no. HGV0000011, http://www.hgvd.genome.med.kyoto-u.ac.jp) for ancient samples and present-day Japanese, respectively.
