# Supplementary material for: Population dynamics in the Japanese Archipelago since the Pleistocene revealed by the complete mitochondrial genome sequences
Source: Sci Rep. 2021 Jun 13;11:12018. doi: 10.1038/s41598-021-91357-2 (PMC8200360; doi:10.1038/s41598-021-91357-2)
Supplement: Supplementary file 1 — Supplementary Information. [file 41598_2021_91357_MOESM1_ESM.pdf]

Population dynamics in the Japanese Archipelago since the Pleistocene revealed by the complete mitochondrial genome sequences

Supplementary Information

Fuzuki Mizuno[1*], Jun Gojobori[2*], Masahiko Kumagai[3], Hisao Baba[4], Yasuhiro Taniguchi[5], Osamu Kondo[6], Masami Matsushita[7], Takayuki Matsushita[7], Fumihiko Matsuda[8], Koichiro Higasa[9], Michiko Hayashi[1], Li Wang[10*], Kunihiko Kurosaki[1], and Shintaroh Ueda[1,6]

1. Department of Legal Medicine, Toho University School of Medicine, Japan
2. Department of Evolutionary Studies of Biosystems, SOKENDAI (The Graduate University for Advanced Studies), Japan
3. Advanced Analysis Center, National Agriculture and Food Research Organization, Japan
4. Department of Anthropology, National Museum of Nature and Science, Japan
5. Department of Archaeology, Faculty of Letters, Kokugakuin University, Japan
6. Department of Biological Sciences, Graduate School of Science, The University of Tokyo, Japan
7. The Organization of Anthropological Research, Japan
8. Graduate School of Medicine, Kyoto University, Japan
9. Department of Genome Analysis, Institute of Biomedical Science, Kansai Medical University, Jap an
10. School of Medicine, Hangzhou Normal University, China

[*]To whom correspondence may be addressed. Email:

fuzuki.mizuno@med.toho-u.ac.jp

gojobori_jun@soken.ac.jp

liwang@hznu.edu.cn

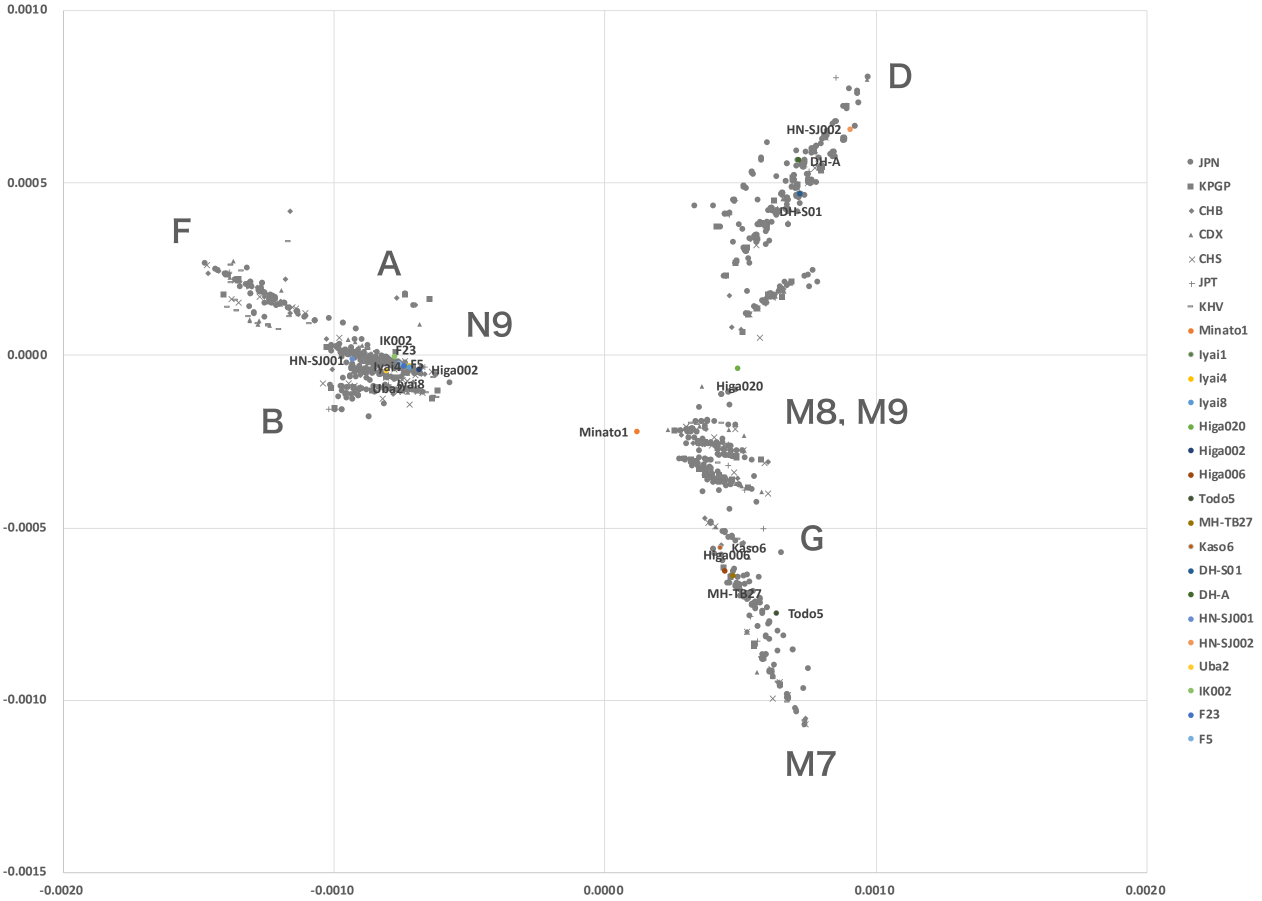

Supplementary Figure S1. Multi Dimensional Scaling (MDS) plot of mitogenome of Minato1, Jomon and Yayoi with present-day individuals from Nagahama study (JPN), EAS of 1000 genome project (CHB, CHS, CDX, JPT and KHV, 1000 Genomes Project Consortium 2015) and KPGP (Kim, J. *et al.* 2020).

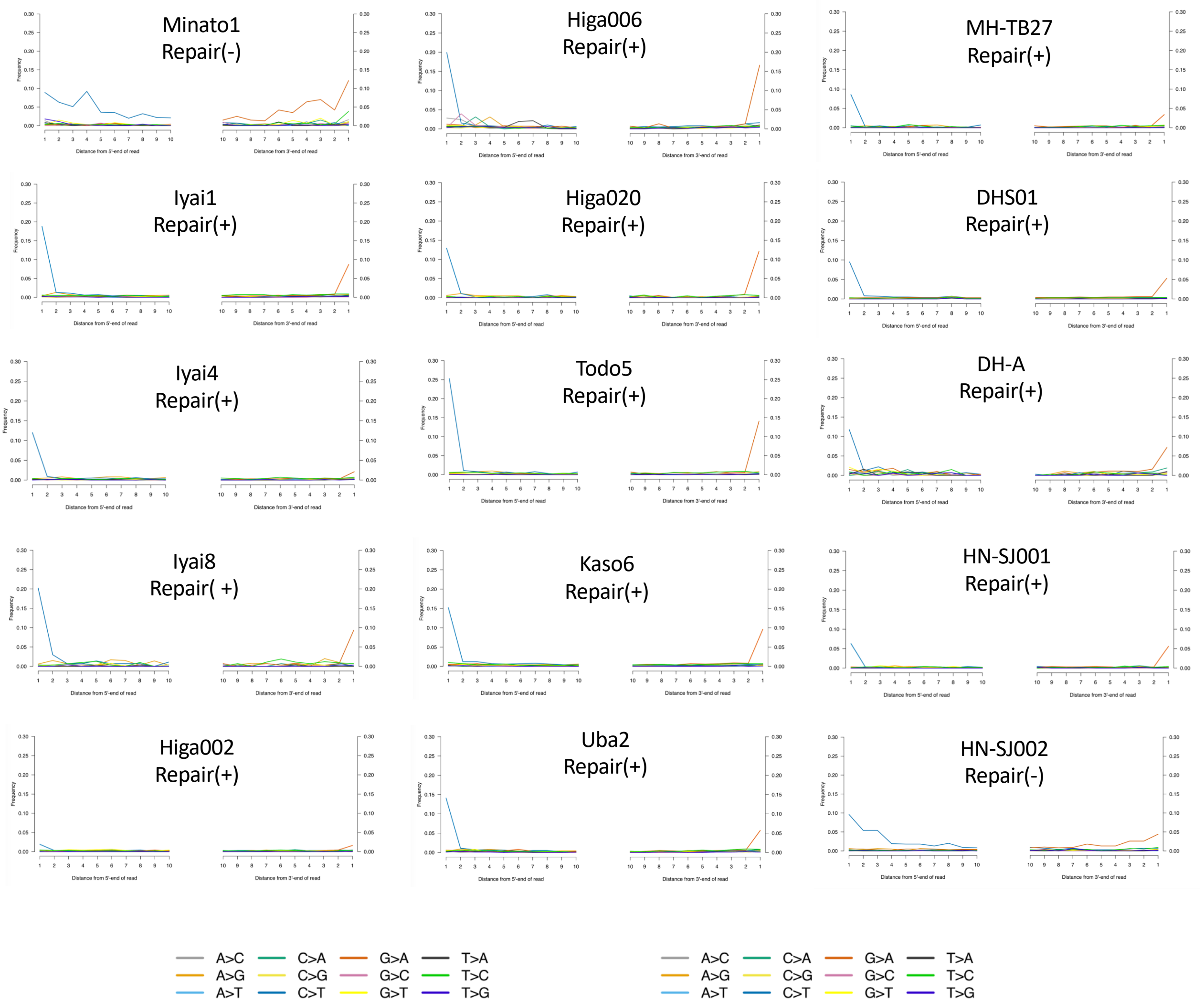

Supplementary Figure S2. Misincorporation plot obtained using MitoSuite.

Misincorporation plot obtained using MitoSuite (Ishiya and Ueda 2017). Postmortem DNA degradation pattern. Nucleotide misincorporation pattern of obtained ancient DNA reads. (Blue) Frequencies of C to T mismatches for the first 10 nucleotides of the 5′-end of paired-end reads. (Red) Frequencies of G to A mismatches for the first 10 nucleotides of the 3′-end of paired-end reads.

Supplementary Table S1. Late Pleistocene and Holocene individuals and their mitogenome results.

ID in this study	Site	Location	Era	Approximate ages	Skeletal element	mtDNA depth (avg.)	mtDNA haplogroup	mtDNA contamination (95% C.I.)#	concordance across mtDNA##
Minato1	Minatogawa fissure	Okinawa Pref.	Paleolithic	19,900 cal BP*	Femur	52	M	1.99-7.30	0.996
Iyai1	Iyai rock-shelter	Gunma Pref.	Initial Jomon	8,300-8,200 cal BP**	Petrous	111	N9b	0.96-5.58	0.994
Iyai4	Iyai rock-shelter	Gunma Pref.	Initial Jomon	8,300-8,200 cal BP**	Petrous	1577	N9b3	1.47-2.66	0.995
Iyai8	Iyai rock-shelter	Gunma Pref.	Initial Jomon	8,300-8,200 cal BP**	Petrous	100	N9b	2.72-7.59	0.992
Higa002	Higashimyou shell midden	Saga Pref.	Initial Jomon	-	Tibia	42	N9a2a	2.50-10.45	0.998
Higa006	Higashimyou shell midden	Saga Pref.	Initial Jomon	7,934-7,792 cal BP***	Tibia	22	M7a1a	0.97-9.55	0.991
Higa020	Higashimyou shell midden	Saga Pref.	Initial Jomon	-	Tibia	11	M80'D	0.46-6.70	0.998
Todo5	Todoroki shell midden	Kumamoto Pref.	Early Jomon	6,210-6,094 cal BP	Petrous	37	M7a1a	3.94-11.95	0.990
Kaso6	Kasori shell midden	Chiba Pref.	Middle Jomon	-	Tooth	127	M7a	3.00-7.67	0.997
Uba2	Ubayama shell midden	Chiba Pref.	Middle Jomon	-	Tooth	44	N9b	1.94-6.94	0.997
MB-TB27	Mabuni hantabaru	Okinawa Pref.	Late Jomon	-	Tibia	15	M7a1a	-1.581-6.581	0.985
DH-S01	Doigahama	Yamaguchi Pref.	Middle Yayoi	2,306-2,238 cal BP	Petrous	5891	D4b2b1	0.34-0.61	0.998
DH-A	Doigahama	Yamaguchi Pref.	Middle Yayoi	-	Tibia	13	D4b2b1	-0.2-2.09	0.997
HN-SJ001	Hanaura	Saga Pref.	Middle Yayoi	-	Tibia	16	B4b1a1a	3.47-10.03	0.998
HN-SJ002	Hanaura	Saga Pref.	Middle Yayoi	-	Tibia	24	D4b2a1	1.03-3.25	0.989

* The currently reported age of Minato 1 was determined from nearby charcoal deposits (Kaifu & Fujita 2011).

** The three human remains were uncovered from individual burial pits, which were close in proximity and from the same layer (Mizuno et al. 2020).

*** Yoneda, M. 2016

mtDNA contamination is based on estimetied mismatch percentages.

The concordance represents consistent base per site of an assembled consensus sequence.

Supplementary Table S2. Haplogroup frequencies of 2,062 present-day Japanese in this study.

Haplogroup	A	B	C	D	F	G	M	N	U	Y	Z
n	155	282	17	787	169	160	385	77	2	3	25
Frequency (%)	7.5	13.7	0.8	38.2	8.2	7.8	18.7	3.7	0.1	0.1	1.2
				D4: 34.3%			M7a: 7.9%	N9b: 1.3%			

Supplementary Table S3. Mitogenome sequences from Present-day Japanese used for construction of Bayesian tree and phylogenetic network.

ID in this study	Haplogroup	Original ID
JPN_B4a1c1a	B4a1c1a	JPN00010
JPN_A5a1a1	A5a1a1	JPN00011
JPN_M7b1a1a	M7b1a1a	JPN00014
JPN_M8a	M8a	JPN00024
JPN_D5a	D5a	JPN00033
JPN_D4b1a1	D4b1a1	JPN00049
JPN_C7a1c	C7a1c	JPN00055
JPN_M7b1a1a1	M7b1a1a1	JPN00063
JPN_D4g1b	D4g1b	JPN00073
JPN_M10a1a1a	M10a1a1a	JPN00075
JPN_N9b	N9b	JPN00089
JPN_D4l1	D4l1	JPN00108
JPN_D4a2a	D4a2a	JPN00110
JPN_B4c1b2a1	B4c1b2a1	JPN00128
JPN_D4b2b	D4b2b	JPN00141
JPN_B4b1	B4b1	JPN00143
JPN_D4b2a	D4b2a	JPN00145
JPN_B4m	B4m	JPN00150
JPN_D4	D4	JPN00152
JPN_D4g1	D4g1	JPN00156
JPN_D4a3b	D4a3b	JPN00181
JPN_Z3	Z3	JPN00195
JPN_F2f	F2f	JPN00210
JPN_B5b	B5b	JPN00214
JPN_B4a1c3b	B4a1c3b	JPN00226
JPN_U4a2	U4a2	JPN00231
JPN_M7c1a3a	M7c1a3a	JPN00237
JPN_G2a1d1	G2a1d1	JPN00248
JPN_Y1a	Y1a	JPN00249
JPN_M7a1	M7a1	JPN00253
JPN_A3	A3	JPN00264
JPN_D4b1a	D4b1a	JPN00265
JPN_D4h1b	D4h1b	JPN00266
JPN_D4a3b1	D4a3b1	JPN00291
JPN_G1a3	G1a3	JPN00308
JPN_D4a3a2	D4a3a2	JPN00311
JPN_B4a3	B4a3	JPN00318
JPN_B5a2a2	B5a2a2	JPN00319
JPN_D4i3	D4i3	JPN00324
JPN_M9a1a1a	M9a1a1a	JPN00325
JPN_N9b1	N9b1	JPN00328
JPN_D5a1a2	D5a1a2	JPN00331
JPN_B4f1	B4f1	JPN00361
JPN_D5c2	D5c2	JPN00365
JPN_G3a2a	G3a2a	JPN00374
JPN_A5c1	A5c1	JPN00376
JPN_M7b1a1a	M7b1a1a	JPN00450
JPN_B4f1	B4f1	JPN00451
JPN_C5	C5	JPN00452
JPN_M7a1a5a	M7a1a5a	JPN00481
JPN_B5a2a	B5a2a	JPN00485
JPN_B5b2c1	B5b2c1	JPN00515
JPN_B4a2b1a	B4a2b1a	JPN00529
JPN_Z5	Z5	JPN00561
JPN_B5a2a1b	B5a2a1b	JPN00572
JPN_B5b1a2	B5b1a2	JPN00590
JPN_A1	A1	JPN00602
JPN_D5b1a	D5b1a	JPN00617
JPN_B	B	JPN00623
JPN_N9a3	N9a3	JPN00626
JPN_G2a1c2	G2a1c2	JPN00634
JPN_B5b2	B5b2	JPN00662
JPN_D4e1	D4e1	JPN00716
JPN_F4a1a	F4a1a	JPN00745
JPN_D4a	D4a	JPN00748
JPN_Z2	Z2	JPN00751
JPN_M7a1a	M7a1a	JPN00754
JPN_M9a1a1	M9a1a1	JPN00764
JPN_D4	D4	JPN00784
JPN_M11b1a1	M11b1a1	JPN00785
JPN_D4h1a1	D4h1a1	JPN00792
JPN_D4b2a2a1	D4b2a2a1	JPN00810
JPN_B4a4	B4a4	JPN00823
JPN_B4f	B4f	JPN00829
JPN_M9a	M9a	JPN00836
JPN_D4e1a2a	D4e1a2a	JPN00873
JPN_M8a3a1	M8a3a1	JPN00889
JPN_M7a1a1	M7a1a1	JPN00894
JPN_F1a3a1	F1a3a1	JPN00914
JPN_D4a1c	D4a1c	JPN00919
JPN_M7a1a3	M7a1a3	JPN00923
JPN_B4a1b1	B4a1b1	JPN00941
JPN_A15	A15	JPN00958

JPN_B4d3a1	B4d3a1	JPN00974
JPN_M7a1b1	M7a1b1	JPN00990
JPN_C1a	C1a	JPN01031
JPN_D4b1b2	D4b1b2	JPN01045
JPN_D4b2b	D4b2b	JPN01046
JPN_F1b1a1a	F1b1a1a	JPN01047
JPN_M7b1a1a1d	M7b1a1a1d	JPN01098
JPN_Y2	Y2	JPN01103
JPN_F1d	F1d	JPN01109
JPN_C7a2	C7a2	JPN01131
JPN_M13a1a	M13a1a	JPN01142
JPN_A5b1a	A5b1a	JPN01188
JPN_M9a	M9a	JPN01194
JPN_D5a2a1a	D5a2a1a	JPN01199
JPN_Y1b1	Y1b1	JPN01240
JPN_F1e1a	F1e1a	JPN01246
JPN_M10a1b	M10a1b	JPN01274
JPN_A5b	A5b	JPN01289
JPN_A8a	A8a	JPN01311
JPN_D4f1	D4f1	JPN01315
JPN_C7a2	C7a2	JPN01323
JPN_D4b2b2	D4b2b2	JPN01327
JPN_G2a5	G2a5	JPN01342
JPN_M7a1a	M7a1a	JPN01370
JPN_D4o1a	D4o1a	JPN01376
JPN_B5b2a1	B5b2a1	JPN01397
JPN_M7a1	M7a1	JPN01414
JPN_N9b3	N9b3	JPN01442
JPN_D4b1b2	D4b1b2	JPN01495
JPN_F1c1a1	F1c1a1	JPN01496
JPN_A5a1a	A5a1a	JPN01503
JPN_B4c1b1	B4c1b1	JPN01532
JPN_G1a1a	G1a1a	JPN01534
JPN_M7a2a	M7a2a	JPN01574
JPN_B4b1b	B4b1b	JPN01600
JPN_G4	G4	JPN01616
JPN_N9b1	N9b1	JPN01620
JPN_B4e	B4e	JPN01621
JPN_B5b	B5b	JPN01627
JPN_D4b1b2	D4b1b2	JPN01634
JPN_F1acf	F1acf	JPN01644
JPN_G2b2	G2b2	JPN01657
JPN_D4	D4	JPN01659
JPN_A5b1a	A5b1a	JPN01665
JPN_D4g2b1a	D4g2b1a	JPN01685
JPN_Z3	Z3	JPN01694
JPN_B4a1c3a	B4a1c3a	JPN01700
JPN_Z4	Z4	JPN01701
JPN_M8a2b	M8a2b	JPN01714
JPN_D4h1c1	D4h1c1	JPN01749
JPN_M10a1a1b1	M10a1a1b1	JPN01752
JPN_M7a2a	M7a2a	JPN01759
JPN_B4c1c1	B4c1c1	JPN01783
JPN_D4m1	D4m1	JPN01786
JPN_N9a1	N9a1	JPN01797
JPN_M7a1a3	M7a1a3	JPN01835
JPN_B4c1a1a1	B4c1a1a1	JPN01841
JPN_M7c1b2b	M7c1b2b	JPN01850
JPN_D5c1a	D5c1a	JPN01851
JPN_D4	D4	JPN01867
JPN_B4d1	B4d1	JPN01875
JPN_D4a3b	D4a3b	JPN01876
JPN_M8a1a	M8a1a	JPN01889
JPN_B4b1a2a	B4b1a2a	JPN01896
JPN_F3b	F3b	JPN01902
JPN_A15b	A15b	JPN01919
JPN_B5b3b	B5b3b	JPN01922
JPN_D4n	D4n	JPN01938
JPN_G2b2b	G2b2b	JPN01939
JPN_A20	A20	JPN01943
JPN_D4b2b1d	D4b2b1d	JPN01956
JPN_N9a3	N9a3	JPN01960
JPN_B4d	B4d	JPN01962
JPN_D4e2	D4e2	JPN01967
JPN_D4b1	D4b1	JPN01983
JPN_D5b2	D5b2	JPN01985
JPN_B4b1c1	B4b1c1	JPN01991
JPN_G4	G4	JPN01995
JPN_D4h1a2	D4h1a2	JPN02001
JPN_N9b	N9b	JPN02028
JPN_B4h	B4h	JPN02031
JPN_F4a1a	F4a1a	JPN02041
JPN_B4c1b	B4c1b	JPN02050
JPN_D4c1a	D4c1a	JPN02051
JPN_N9a2a	N9a2a	JPN02077
JPN_F1a1b	F1a1b	JPN02092
JPN_Z3d	Z3d	JPN02093
JPN_B4b1a1a	B4b1a1a	JPN02107

Supplementary Table S4. Mitogenome sequences used in the phylogenetic network.

Original ID in the reference	Accession	Haplogroup	Population	Reference	
IK002	-	N9b1	Ancient Jomon	1	
-	KC417443.1	B	Ancient Chinese	2	
F5	-	N9b1	Ancient Jomon	3	
F23	-	N9b1	Ancient Jomon	3	
Brn002	MH359190.1	D2ab	Ancient Trans-Baikal Neolithic	4	*
Brn001	MH359191.1	A	Ancient Trans-Baikal Mesolithic	4	*
Brn003	MH359192.1	D4	Ancient Trans-Baikal Neolithic	4	*
Brn008	MH359193.1	D4	Ancient Trans-Baikal Mesolithic	4	*
irk00x	MH359197.1	C5c	Ancient Trans-Baikal Mesolithic	4	*
irk032	MH359202.1	D6c	Ancient Cis-Baikal Medieval	4	*
irk040	MH359206.1	A	Ancient Cis-Baikal Neolithic	4	*
Irk051	MH359208.1	F1b1f	Ancient Cis-Baikal Mesolithic	4	*
Irk068	MH359212.1	F1b1b	Ancient Cis-Baikal Neolithic	4	*
mak001	MH359219.1	D4	Ancient Cis-Baikal Neolithic	4	*
N3a	MH359221.1	C4b3	Ancient Yakutia Iron Age	4	*
N5a	MH359224.1	D3	Ancient Yakutia Middle Neolithic	4	*
yak021	MH359225.1	C4b	Ancient Yakutia Late Neolithic	4	*
yak022	MH359226.1	C4b1	Ancient Yakutia Late Neolithic	4	*
yak025	MH359229.1	R1	Ancient Yakutia Paleolithic	4	*
GoyetQ116-1	KU534952.1	M	Ancient Europe	5	
GoyetQ376-3	KU534953.1	M	Ancient Europe	5	
LaRochette	KU534951.1	M	Ancient Europe	5	
HG00419	-	B4g2	Southern Han Chinese	6	**
HG00443	-	M8a2a1	Southern Han Chinese	6	**
HG00446	-	B4b1	Southern Han Chinese	6	**
HG00457	-	M33c	Southern Han Chinese	6	**
HG00533	-	B4d1'2'3	Southern Han Chinese	6	**
HG00559	-	M10a1b	Southern Han Chinese	6	**
HG00581	-	D4j15	Southern Han Chinese	6	**
HG00614	-	Z	Southern Han Chinese	6	**
HG00622	-	G2a'c	Southern Han Chinese	6	**
HG00623	-	B4c1b2a2	Southern Han Chinese	6	**
HG00626	-	M12a1a1	Southern Han Chinese	6	**
HG00650	-	M7b1a1f	Southern Han Chinese	6	**
HG00653	-	N8	Southern Han Chinese	6	**
HG00654	-	B4h	Southern Han Chinese	6	**
HG00663	-	R9b1	Southern Han Chinese	6	**
HG00683	-	M71a1a	Southern Han Chinese	6	**
HG00708	-	B5a1c	Southern Han Chinese	6	**
HG00864	-	C7a1	Chinese Dai in Xishuangbanna	6	**
HG01597	-	B	Kinh in Ho Chi Minh City	6	**
HG01795	-	M61	Chinese Dai in Xishuangbanna	6	**
HG01796	-	R9b1a3	Chinese Dai in Xishuangbanna	6	**
HG01797	-	F3a1	Chinese Dai in Xishuangbanna	6	**
HG01799	-	B4c2	Chinese Dai in Xishuangbanna	6	**
HG01812	-	M12a1a	Chinese Dai in Xishuangbanna	6	**
HG01851	-	M7c2	Kinh in Ho Chi Minh City	6	**
HG01872	-	B4	Kinh in Ho Chi Minh City	6	**
HG02016	-	M29Q	Kinh in Ho Chi Minh City	6	**
HG02020	-	R9c	Kinh in Ho Chi Minh City	6	**
HG02028	-	M74a	Kinh in Ho Chi Minh City	6	**
HG02049	-	B4c2	Kinh in Ho Chi Minh City	6	**
HG02064	-	B4b1a	Kinh in Ho Chi Minh City	6	**
HG02070	-	M51a2	Kinh in Ho Chi Minh City	6	**
HG02116	-	C4d	Kinh in Ho Chi Minh City	6	**
HG02122	-	B4a	Kinh in Ho Chi Minh City	6	**
HG02152	-	D5b3	Chinese Dai in Xishuangbanna	6	**
HG02164	-	B6a	Chinese Dai in Xishuangbanna	6	**
HG02165	-	B5a1a	Chinese Dai in Xishuangbanna	6	**
HG02184	-	B4	Chinese Dai in Xishuangbanna	6	**
HG02356	-	M71a	Chinese Dai in Xishuangbanna	6	**
HG02373	-	R11b1	Chinese Dai in Xishuangbanna	6	**
HG02375	-	F3b	Chinese Dai in Xishuangbanna	6	**
HG02377	-	M20	Chinese Dai in Xishuangbanna	6	**
HG02383	-	D4g2a1b	Chinese Dai in Xishuangbanna	6	**
HG02394	-	F1e3	Chinese Dai in Xishuangbanna	6	**
NA18532	-	D4m2a	Han Chinese in Beijing	6	**
NA18534	-	F1e2	Han Chinese in Beijing	6	**

NA18539	-	K3	Han Chinese in Beijing	6	**
NA18541	-	B4i1	Han Chinese in Beijing	6	**
NA18542	-	C7a	Han Chinese in Beijing	6	**
NA18544	-	B4d1a	Han Chinese in Beijing	6	**
NA18548	-	C4a1a2	Han Chinese in Beijing	6	**
NA18550	-	M7b1a1c	Han Chinese in Beijing	6	**
NA18552	-	B4c1b2c	Han Chinese in Beijing	6	**
NA18553	-	N10a	Han Chinese in Beijing	6	**
NA18566	-	C5	Han Chinese in Beijing	6	**
NA18592	-	F4b	Han Chinese in Beijing	6	**
NA18608	-	M11c	Han Chinese in Beijing	6	**
NA18609	-	F2c2	Han Chinese in Beijing	6	**
NA18614	-	B4h1	Han Chinese in Beijing	6	**
NA18629	-	F2e	Han Chinese in Beijing	6	**
NA18632	-	A15	Han Chinese in Beijing	6	**
NA18634	-	G1	Han Chinese in Beijing	6	**
NA18637	-	G2b1a2	Han Chinese in Beijing	6	**
NA18942	-	Z4a1a1	Japanese in Tokyo	6	**
NA19011	-	M7a1	Japanese in Tokyo	6	**
NA19072	-	Z3c	Japanese in Tokyo	6	**
-	EF153779.1	C1a	Buryat	7	
-	EF153781.1	M7a2	Buryat	7	
-	EF153782.1	M7c2a	Buryat	7	
-	EF153790.1	M7c2a	Buryat	7	
-	EF153791.1	A4	Buryat	7	
-	EF153792.1	A4a1	Buryat	7	
-	EF153793.1	X2e	Buryat	7	
-	EF153794.1	A4	Buryat	7	
-	EF153795.1	A4c	Buryat	7	
-	EF153796.1	D2	Buryat	7	
-	EF153797.1	A8	Buryat	7	
-	EF153798.1	Y2	Buryat	7	
-	EF153799.1	A4a1	Buryat	7	
-	EF153800.1	D3	Buryat	7	
-	EF153801.1	A5c	Buryat	7	
-	KC993933.1	B4b1a2	Aeta	8	
-	KC993934.1	P	Aeta	8	
-	KC993935.1	P	Aeta	8	
-	KC993937.1	M7b3a	Aeta	8	
-	KC993939.1	B4b1a	Aeta	8	
-	KC993941.1	B4b1a2	Aeta	8	
-	KC993942.1	P	Aeta	8	
-	KC993943.1	B4b1a2	Aeta	8	
-	KC993946.1	B4b1a2	Aeta	8	
-	KC993954.1	F1a3a	Aeta	8	
Mam37	GU733723.1	N11b	Mamanwa	9	
Mam38	GU733724.1	B4b	Mamanwa	9	
Mam51	GU733734.1	M74b	Mamanwa	9	
Mam56	GU733737.1	M7c	Mamanwa	9	
Mam57	GU733738.1	R9b	Mamanwa	9	
Mam61	GU733741.1	B4b	Mamanwa	9	
Mam64	GU733744.1	E2a	Mamanwa	9	
MA101	MG672483.1	B4b	Mlabri	10	
MAN10	MG672501.1	B5a1b1	Manobo	10	
MAN11	MG672502.1	M17	Manobo	10	
MAN12	MG672503.1	R21	Manobo	10	
MAN13	MG672504.1	M21a	Manobo	10	
MAN14	MG672505.1	M21a	Manobo	10	
KPGP-00032	-	A12	Korean	11	**
KPGP-00056	-	Z3	Korean	11	**
KPGP-00229	-	M11	Korean	11	**
KPGP-00266	-	C4a2a	Korean	11	**
KPGP-00326	-	D6a1	Korean	11	**
KPGP-00321	-	B4b1a2	Korean	11	**
KPGP-00336	-	R11b	Korean	11	**
KPGP-00340	-	KOR2_R11b	Korean	11	**
KPGP-00351	-	M9a4b	Korean	11	**
KPGP-00349	-	J1c2	Korean	11	**
KPGP-00350	-	C4a	Korean	11	**
KPGP-00347	-	D4b1a	Korean	11	**

* The gap sequences in these sequenes were imputated by the method of reference number 12.

** These mtDNA sequences are obtained by the method of reference number 13 using mapped reads to chrM of corresponding bam file.

SI References

Supplementary Figure S1 References

1. 1000 Genomes Project Consortium. A global reference for human genetic variation. Nature **526**, 68-74 (2015).
2. Kim, J. *et al*. The origin and composition of Korean ethnicity analyzed by ancient and present-day genome sequences. Genome Biology and Evolution **12**, 553-565 (2020).

Supplementary Figure S2 Reference

1. Ishiya, K & Ueda, S. MitoSuite: A graphical tool for human mitochondrial genome profiling in massive parallel sequencing. *PeerJ* **5,** e3406 (2017).

Supplementary Table S1 References:

1. Kaifu, Y. & Fujita, M. Fossil record of early modern humans in east Asia. *Quaternary International* **248,** 2-11 (2012).
2. Kondo, O. *et al.* A female human skeleton from the Initial Jomon period found in the Iyai rock shelter in mountainous Kanto, Japan. *Anthropological Science* **126,** 151-164 (2018).
3. Mizuno, F. *et al.* A study of 8,300-year-old Jomon human remains in Japan using complete mitogenome sequences obtained by next-generation sequencing. *Annals of Human Biology* **47,** 555-559 (2020).
4. Yoneda, M. Higashimyo isekigun IV *Higashimyo isekigun soukatsuhoukokusyo*, (in Japanese) **1,** 68-71 (2016).

Supplementary Table S4 References

1. McColl, H. *et al*. The prehistoric peopling of southeast Asia. *Science* **361,** 88-92 (2018).
2. Fu, Q. *et al.* DNA analysis of an early modern human from Tianyuan cave, China. *Proceedings of the National Academy of Sciences of the United States of America* **110,** 2223-2227 (2013).
3. Kanzawa-Kiriyama, H. *et al.* Late Jomon male and female genome sequences from the Funadomari site in Hokkaido, Japan. *Anthropological Science* **127,** 83-108 (2019).
4. Kılınç, G.M. *et al.* Investigating Holocene human population history in north Asia using ancient mitogenomes. *Scientific Reports* **8,** 8969 (2018).

5. Posth, C. *et al*. Pleistocene mitochondrial genomes suggest a single major dispersal of non-Africans and a late glacial population turnover in Europe. *Current Biology* **26,** 827-833 (2016).
6. 1000 Genomes Project Consortium. A global reference for human genetic variation. *Nature* **526,** 68-74 (2015).
7. Derenko, M. *et al*. Phylogeographic analysis of mitochondrial DNA in northern Asian populations. *American Journal of Human Genetics* **81,** 1025-1041 (2007).
8. Delfin, F. *et al*. Complete mtDNA genomes of Filipino ethnolinguistic groups: a melting pot of recent and ancient lineages in the Asia-Pacific region. *European Journal of Human Genetics* **22,** 228-237 (2014).
9. Gunnarsdóttir, E.D. *et al.* High-throughput sequencing of complete human mtDNA genomes from the Philippines. *Genome Research* **21,** 1-11 (2011).
10. Kutanan, W. *et al*. Contrasting maternal and paternal genetic variation of hunter-gatherer groups in Thailand. *Scientific Reports* **8,** 1536 (2018).
11. Kim, J. *et al.* The origin and composition of Korean ethnicity analyzed by ancient and present-day genome sequences. *Genome Biology and Evolution* **12,** 553-565 (2020).
12. Ishiya, K. *et al.* MitoIMP: A computational framework for imputation of missing data in low-coverage human mitochondrial genome. *Bioinformatics and Biology Insights* **13,** 1177932219873884 (2019).
13. Ishiya, K & Ueda, S. MitoSuite: A graphical tool for human mitochondrial genome profiling in massive parallel sequencing. *PeerJ* **5,** e3406 (2017).
